# Development of a recombinase-aided amplification combined with a lateral flow dipstick assay for rapid detection of H7 subtype avian influenza virus

**DOI:** 10.3389/fmicb.2023.1286713

**Published:** 2023-11-03

**Authors:** Fuyou Zhang, Jiajing Shang, Juan Luo, Xin Yin, Xiaohui Yu, Wenming Jiang, Jinping Li, Liping Yuan, Guangyu Hou, Hualei Liu, Yang Li

**Affiliations:** ^1^China Animal Health and Epidemiology Center, Qingdao, China; ^2^Hebei University of Engineering, Handan, China

**Keywords:** avian influenza virus, reverse transcription recombinase-aided amplification assay, lateral flow dipstick, sensitivity, specificity, on-site inspection

## Abstract

Avian influenza viruses (AIV) pose a significant persistent threat to the public health and safety. It is estimated that there have been over 100 outbreaks caused by various H7 subtypes of avian influenza viruses (AIV-H7) worldwide, resulting in over 33 million deaths of poultry. In this study, we developed a recombinase-aided amplification combined with a lateral flow dipstick assay for the detection of hemagglutinin (HA) genes *to provide technical support for* rapid clinical detection of AIV-H7. The results showed that the assay can complete the reaction within 30 min at a temperature of 39°C. Specificity tests demonstrated that there was no cross-reactivity with other common poultry pathogens, including Newcastle disease virus (NDV) and infections bronchitis virus (IBV). The detection limit of this assay was 1 × 10^1^ copies/μL, while RT-qPCR method was 1 × 10^1^ copies/μL, and RT-PCR was 1 × 10^2^ copies/μL. The κ value of the RT-RAA-LFD and RT-PCR assay in 132 avian clinical samples was 0.9169 (*p* < 0.001). These results indicated that the developed RT-RAA-LFD assay had good specificity, sensitivity, stability and repeatability and may be used for rapid detection of AIV-H7 in clinical diagnosis.

## 1. Introduction

Influenza A viruses can infect both birds and mammals, posing a potential threat to human health and safety (Yang et al., 2018; He et al., 2020) and causing significant economic losses to the poultry industry. Avian influenza virus (AIV), a member of the influenza A virus that cause a range of symptoms, from respiratory issues to severe systemic sepsis (Zhou P. et al., 2017). Based on its pathogenicity in chickens, the virus can be categorized as high pathogenicity avian influenza virus (HPAIV) and low pathogenicity avian influenza virus (LPAIV) (Fereidouni et al., 2010; Swayne et al., 2012; Tang et al., 2021). HPAI is a legally recognized disease by the World Organization for Animal Health (WOAH) and is categorized as a Class I animal disease by the Ministry of Agriculture and Rural Affairs of China.

Influenza viruses consist of eight gene segments among which HA and NA genes are critical factors influencing the pathogenicity, transmission, and host specificity of the virus. The antigenicity of these genes is also the primary basis for virus typing. Normally, AIV recognizes α-2, 3 sialic acid receptors found specifically in birds, while human influenza viruses recognize α-2, 6 sialic acid receptors. As a result, AIV does not easily infect humans or cause the spread of disease in humans. However, some mammalian respiratory epithelial cells possess α-2, 3 sialic acid receptors, which allows AIV to infect them. This process has led to the gradual evolution of AIV strains that can infect humans. Furthermore, AIV is highly susceptible to mutation, frequently occurring during replication and readily inserting or recombining genes with other influenza viruses. Currently, the H7N9 subtype is one of the primary epidemic strains in China, with occasional appearances of other subtypes such as H7N8 and H7N3. Since the first report of human infection with AIV-H7N9 in China in 2013 (Gao et al., 2013), H7N9 has caused at least five epidemics resulting in over 1,500 infections and more than 600 deaths (Iuliano et al., 2017; Wang et al., 2017). Studies have demonstrated that compared to poultry, only two gene fragments of H7N9 found in Chinese patients are derived from poultry H7N9, while the other six gene fragments have originated from H9N2. In addition to reassortment with endemic influenza H9N2 (Ke et al., 2014; Liu et al., 2014; Qi et al., 2014; Zhou L. et al., 2017; Quan et al., 2018; Bai et al., 2020), H7N9 can also recombine with seasonal influenza H3N2 (Gao et al., 2013; Li et al., 2014; Pu et al., 2019), H5N6 (Jin et al., 2017) or even H7N9 (Ma et al., 2018) itself.

Various methods have been developed to detect AIV-H7, including viral culture and isolation, enzyme-linked immunosorbent assay (ELISA) (Jensen et al., 2013; Dong et al., 2019; Li G. et al., 2021; Li Y. et al., 2021), reverse transcription-polymerase chain reaction (RT-PCR) (Li et al., 2018; Liu et al., 2018; Yang et al., 2022), reverse transcription loop-mediated isothermal amplification (RT-LAMP) (Bao et al., 2012) and other novel methods (Huang et al., 2016; Xiao et al., 2019; Xiang et al., 2022). However, many of these methods have limitations such as longer reaction times, high operational requirements, and the need for laboratory facilities. Furthermore, most of these methods require specialized equipment and are not suitable for on-site detection. Therefore, there is a need to develop a stable and reliable method that can rapidly detect AIV-H7 on-site to aid in the early prevention and control of avian influenza.

reverse transcription recombinase-aided amplification combined with a lateral flow dipstick (RT-RAA-LFD) is a novel and innovative nucleic acid detection technology that combines RAA and LFD methods to rapidly, conveniently, and accurately detect target DNA sequences. This method involves amplifying the target DNA sequence using the RAA method, and then visualizing the results using LFD technology once the amplified product is combined with a labeled probe. In this study, an RT-RAA-LFD approach was established with advantages in respect to sensitivity, specificity, rapidity, and simplicity which making it suitable for rapid on-site testing.

## 2. Materials and methods

### 2.1. Samples collection and nucleic acid extraction

All clinical samples, including throat and cloacal swabs, used in this study were collected and stored by China Animal Health and Epidemiology Center. Previous studies in our laboratory have validated Newcastle disease virus (NDV), Infectious bronchitis virus (IBV), and Avian influenza virus (AIV) by RT-PCR and gene sequencing. Samples were stored in Phosphate Buffered Saline (PBS) containing penicillin–streptomycin (penicillin: 4,000 IU/mL, streptomycin: 4 μg/mL) and centrifuged at 12,000 *r/min*. The supernatant from each sample was used for RNA extraction with the Pathogen 96 QIA cube HT kit (Qiagen, Germany). The extracted RNA was stored at −80°C for subsequent experiments.

### 2.2. Design of primers and probes

A total of 87 full-length sequence of the HA gene for the major circulating strains of H7 has been downloaded from the GenBank and GISAID databases. After the alignment of the sequence using MEGA 7, six pairs of primers and one probe were designed for the conserved region of the HA gene using Oligo 7 according to the RAA primer and probe design principle to ensure their effectiveness. Biotin labeling was added to the reverse primer at the 5′ end, while the probe was labeled with FAM fluorescence at the 5′ end and C3 Spacer modifications at the 3′ end. The 32rd base was replaced with tetrahydrofuran (THF). All the primers and probes were validated and optimized by RT-RAA and were synthesized by Sangon Biotech (Shanghai, China). The details of the primers and probes used for RT-RAA amplification are listed in Table 1.

**Table 1:** Primers and probe sequences used for this study.

Primer	Sequences (5′-3′)	Size (bp)	Gene	Source
F1	CGGACTGCGAGAGGCCTATTTGGTGCTATA	30	HA	This study
R1	Biotin-GATTGAGTGCTTTTGTAATCTGCAGCAGTT	30	HA	This study
F2	GAGAAAACGGACTGCGAGAGGCCTATTTGG	30	HA	This study
R2	Biotin-CTATCGCCGATTGAGTGCTTTTGTAATCTG	30	HA	This study
F3	GTTCCAAAGAGAAAACGGACTGCGAGAGGC	30	HA	This study
R3	Biotin-CTTTTGTAATCTGCAGCAGTTCCCTCTC	28	HA	This study
F4	GCGAGAGGCCTATTTGGTGCTATAGCGGG	29	HA	This study
R4	Biotin-GTAATCTGCAGCAGTTCCCTCTCCCTGTG	29	HA	This study
F5	GAGGCCTATTTGGTGCTATAGCGGGGTTC	29	HA	This study
R5	Biotin-CAGCAGTTCCCTCTCCCTGTGCATTTTG	28	HA	This study
F6	GCCTATTTGGTGCTATAGCGGGGTTCATTG	30	HA	This study
R6	Biotin-CAGTTCCCTCTCCCTGTGCATTTTGATGTC	30	HA	This study
P	FAM-TTCATTGAAAATGGATGGGAAGGCCTAAT TG/THF/TGGTTGGTATGGTTTC [C3 Spacer]	49	HA	This study
HA-F	TATTCGTCTCAGGGAGCAAAAGCAGGGG	28	HA	Hoffmann et al. (2001)
HA-R	ATATCGTCTCGTATTAGTAGAAACAA GGGTGTTTT	35	HA	Hoffmann et al. (2001)
H7-F	CTAATTGATGGTTGGTATGGTTTCA	25	HA	GB/T 18936—2020
H7-R	AATTGCCGATTGAGTGCTTTT	21	HA	GB/T 18936—2020
H7-P	FAM-CAGAATGCACAGGGAGAGGGAACTGCT-BHQ1	27	HA	GB/T 18936—2020

### 2.3. Preparation of standard sample

In this study, the full length of the HA gene of AIV-H7 was amplified using a One-step RT-PCR kit (Vazyme, Nanjing, China). The amplified HA gene fragment was then recovered using the gel recovery kit (Takara, Shanghai, China) and attached to the T carrier. After cutting the plasmid of the positive bacteria into single strands, the fragment of interest was transcribed into RNA using the T7 transcription kit (Promega, United States). The RNA was then purified using the SanPrep Column Plasmid Mini-Preps Kit (Sangon, Shanghai, China). RNA quantification was performed using the Thermo Fisher Scientific Nanodrop Onec Trace Nucleic Acid Protein Concentration Analyzer (Thermo Fisher Scientific, United States). The copy numbers of the *in vitro* RNA transcripts were calculated according to the following formula: RNA quantity (copies/μL) = RNA concentration (ng/μL × 10^−9^) × (6.02 × 10^23^) / (DNA length × 340) The constructed standards were diluted to a concentration gradient of 10^9^ to 10^0^ copies/μL using the 10-fold dilution method and stored at −80°C for later use.

### 2.4. Establishment of RT-RAA-LFD method

The RT-RAA assay was performed using a commercial RT-RAA kit (Qitian, China) in a reaction volume of 50 μL. The reaction mixtures contained 25 μL of reaction buffer, 16.7 μL ddH_2_O, 0.6 μL of target-specific RT-TAA probe, 2.1 μL of the forward primer (10 μM), 2.1 μL of the biotin-labeled reverse primer (10 μM), 1 μL RNA template, and 2.5 μL of 280 mM magnesium acetate, which was pipetted into the tube lids. After all the reaction components were added, the mixture was vortexed and centrifuged at 39°C in an oscillating mixer (Qitian, China). The amplification was performed at 39°C for 20 min, using an RT-RAA fluorescence detection equipment (QT-RAA-F7200; Qitian, Jiangsu, China). The amplification product was then diluted 50 times with PBS and tested using commercial lateral flow dipsticks (Warbio, Nanjing, China). After a few minutes, the results can be observed. A positive result was indicated by both the test line and control line appearing simultaneously, while only the control line appearing indicated a negative result. A doubtful result was indicated by only the test line appearing, and a retest was necessary.

### 2.5. Optimization of RT-RAA-LFD reaction conditions

Reaction temperature and reaction time are important issues which influence the efficiency of the amplification efficiency of the RT-RAA-LFD assay. In order to optimize the system, the reaction temperature was set to 37°C, 39°C, and 41°C, respectively, and the reaction time was set to 15, 20, and 25 min, respectively. Optical images of LFD were taken using a Canon EOS 6D digital camera (Tokyo, Japan), and converted into 8-bit grayscale format by Image J (National Institutes of Health (NIH), Bethesda, MD, United States). The background signal of the images was eliminated, and the optical density of the selected T and C lines was automatically measured. The relative densitometric ratio between the T line and the C line (T/C ratio) was calculated for each LFD. The optimal reaction temperature was determined by the temperature corresponding to the amplification product with the strongest T-line signal intensity and the highest T/C ratio. The shortest reaction time that produced the strongest T-line signal intensity and the highest T/C ratio at the optimal temperature was deemed the optimal reaction time.

### 2.6. Analytical specificity of RAA-LFD

The RNA of NDV, IBV, AIV-H3, AIV-H5, AIV-H7, and AIV-H9 were used as templates for the optimized RT-RAA-LFD assay, while ddH_2_O was used as a negative control. These viruses, previously identified by our laboratory and listed in Table 2, were used to determine the analytical specificity of the assay.

**Table 2:** Some information about the samples used for analytical specificity in the study.

Sample	Virus	H7 RT-PCR assay	H7 RT-qPCR assay
E2015	NDV	−	−
X2308	IBV	−	−
E2167	H3N8	−	−
A2175	H5N1	−	−
J2341	H7N9	+	+
G2257	H9N2	−	−

+: positive; −: negative.

### 2.7. Analytical sensitivity of RT-RAA-LFD

To assess and compare the sensitivity of RT-RAA-LFD, RT-qPCR, and RT-PCR, serial dilutions of RNA standards ranging from 10^5^ to 10^0^ copies/μL were used as templates. The optimized conditions for the RT-RAA-LFD assay utilized in the sensitivity test. The One-Step RT-qPCR Kit (Takara, Beijing, China) was employed for the RT-qPCR assay. The reaction system (25 μL) contained 12.5 μL of qPCR Mix, 1 μL each of forward (10 μM) and reverse (10 μM) primers, 8.5 μL of ddH_2_O, and 2 μL of the template. The reaction solution was pre-denatured at 95°C for 30 s, denatured at 95°C for 15 s, and annealed at 58°C for 5 s, resulting in a total of 40 cycles. The One-step RT-PCR Kit (Vazyme, Nanjing, China) was used for the RT-PCR assay. The sensitivity tests of the qPCR and RT-PCR were conducted three independent times. Meanwhile, eight replicates for each dilution gradient of the selected standard samples were then tested by the RT-RAA-LFD method.

### 2.8. Detection of clinical samples

To evaluate the agreement between RT-RAA-LFD, RT-qPCR, and RT-PCR, a total of 132 throat and cloacal swab samples collected from live poultry markets were detected. Kappa statistics and *P* were assessed for agreement between assays on different specimens. Kappa is a statistical measure of inter-rater agreement, and its value ranges between −1 ~ 1. A value of 1 indicates complete agreement, while a value of 0 indicates agreement due to chance and a value of <0 indicates disagreement. *P* is the probability of the observed agreement occurring by chance.

## 3. Results

### 3.1. Design and screening of the primer-probe combinations

**Figure 1:**
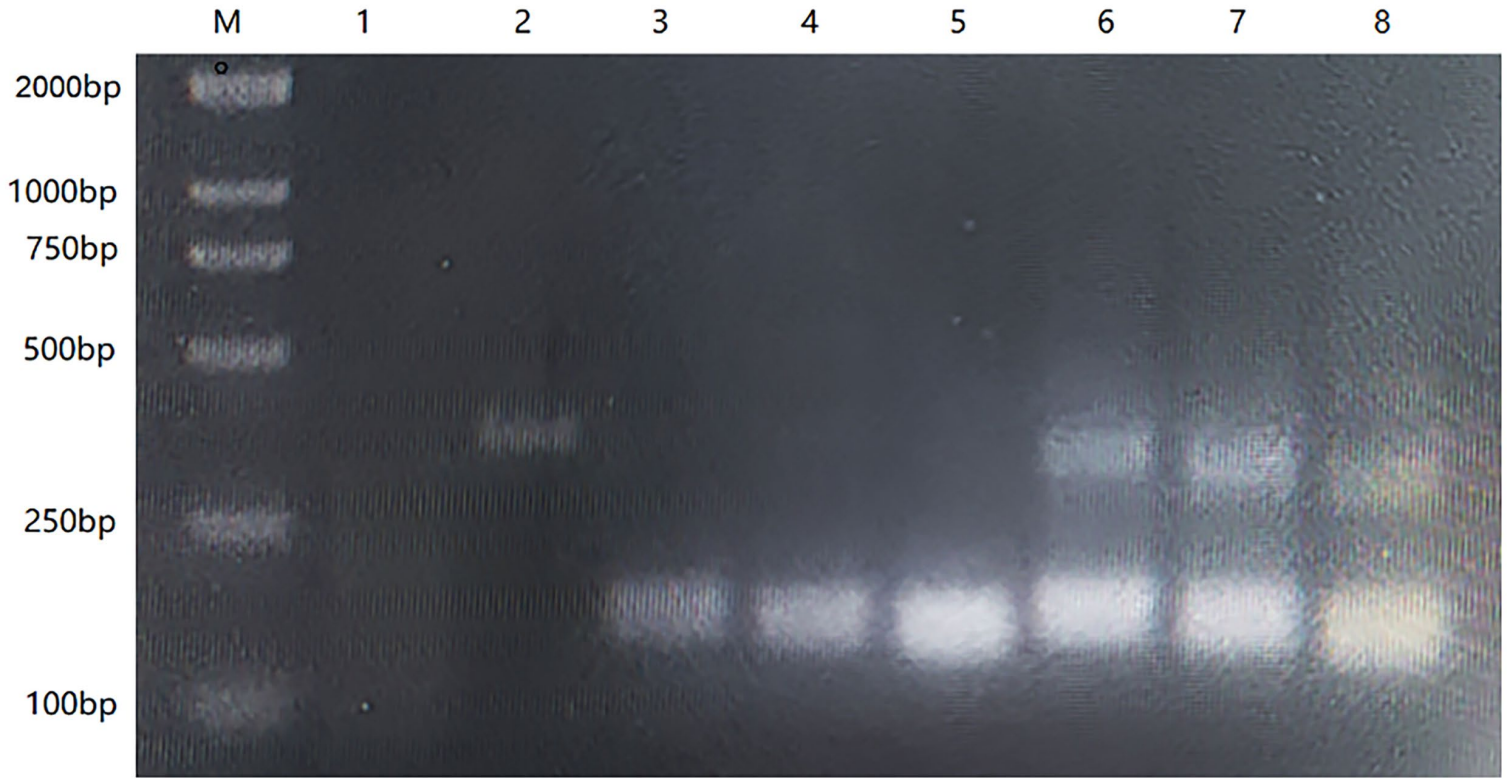
The result of 1% agarose gel electrophoresis screening primers with RT-PCR. M: DL2000 Maker; 1: Negative control; 2: Positive control; 3–8: F1/R1, F2/R2, F3/R3, F4/R4, F5/R5, F6/R6.

We aligned the full-length HA gene of the AIV-H7 subtype reported in recent years and selected a highly conserved fragment. Based on the RAA primer and probe design principles, we successfully designed the probe with the highest score and the primers surrounding the probe. To determine the optimal primer combination, we performed an RAA amplification using a commercial RAA kit (Qitian, Jiangsu, China) as previously described. We used agarose gel electrophoresis to demonstrate the RAA results, and the primer-probe combination that produced a single and bright band was deemed optimal. The results exhibited positive amplification for all primer combinations, with F1/R1, F2/R2, and F3/R3 producing a single band, while F4/R4, F5/R5, and F6/R6 showed non-specific amplifications (Figure 1). Among the primer combinations that generated a single band, the F3/R3 amplification band was clearer and brighter, thus making F3/R3 the selected primer combination for subsequent experiments.

### 3.2. Optimization of reaction conditions

**Figure 2:**
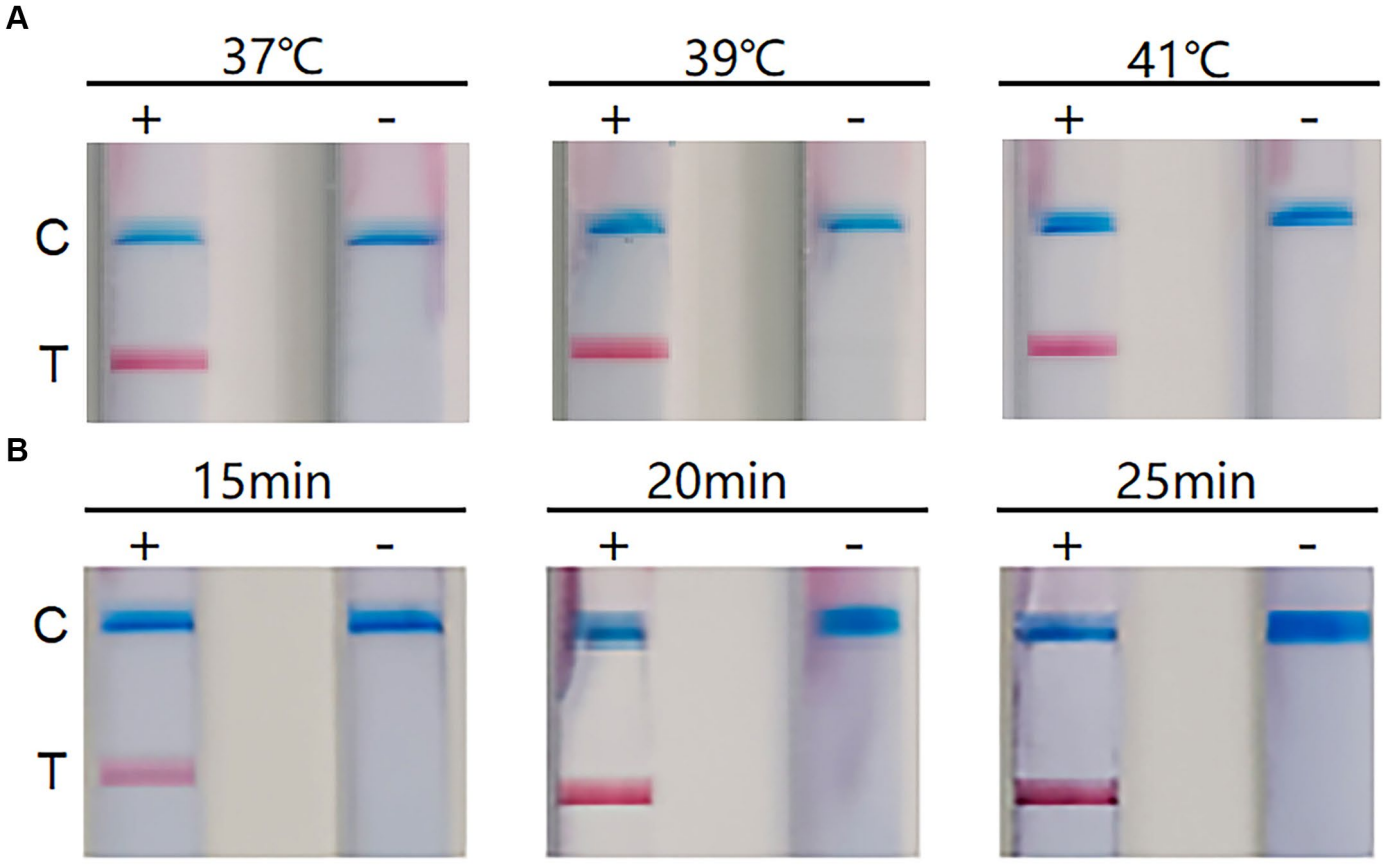
The results of reaction conditions. **(A)** Optimization of reaction temperature. +: positive; −: negative; C: Control line; T: Test line. **(B)** Optimization of reaction time. +: positive; −: negative; C: Control line; T: Test line.

The RT-RAA-LFD reaction could be performed within a certain range of reaction temperatures and times. To determine the optimum temperature and reaction time, we tested the reaction temperature range of 37 ~ 41°C and found no significant difference in the clarity and brightness of the bands (Figure 2A). Therefore, we selected 39°C as the reaction temperature, based on the requirements of the RT-RAA kit (Qitian, Jiangsu, China). Next, we optimized the reaction time and tested 15 min, 20 min, and 25 min as the possible reaction times. The results indicated no significant difference in the C line at different times, but at 20 min, the T line appeared cleaner and brighter (Figure 2B). Therefore, the optimum reaction temperature was 39°C and the reaction time was 20 min.

### 3.3. The analytical specificity of the RT-RAA-LFD assay

**Figure 3:**
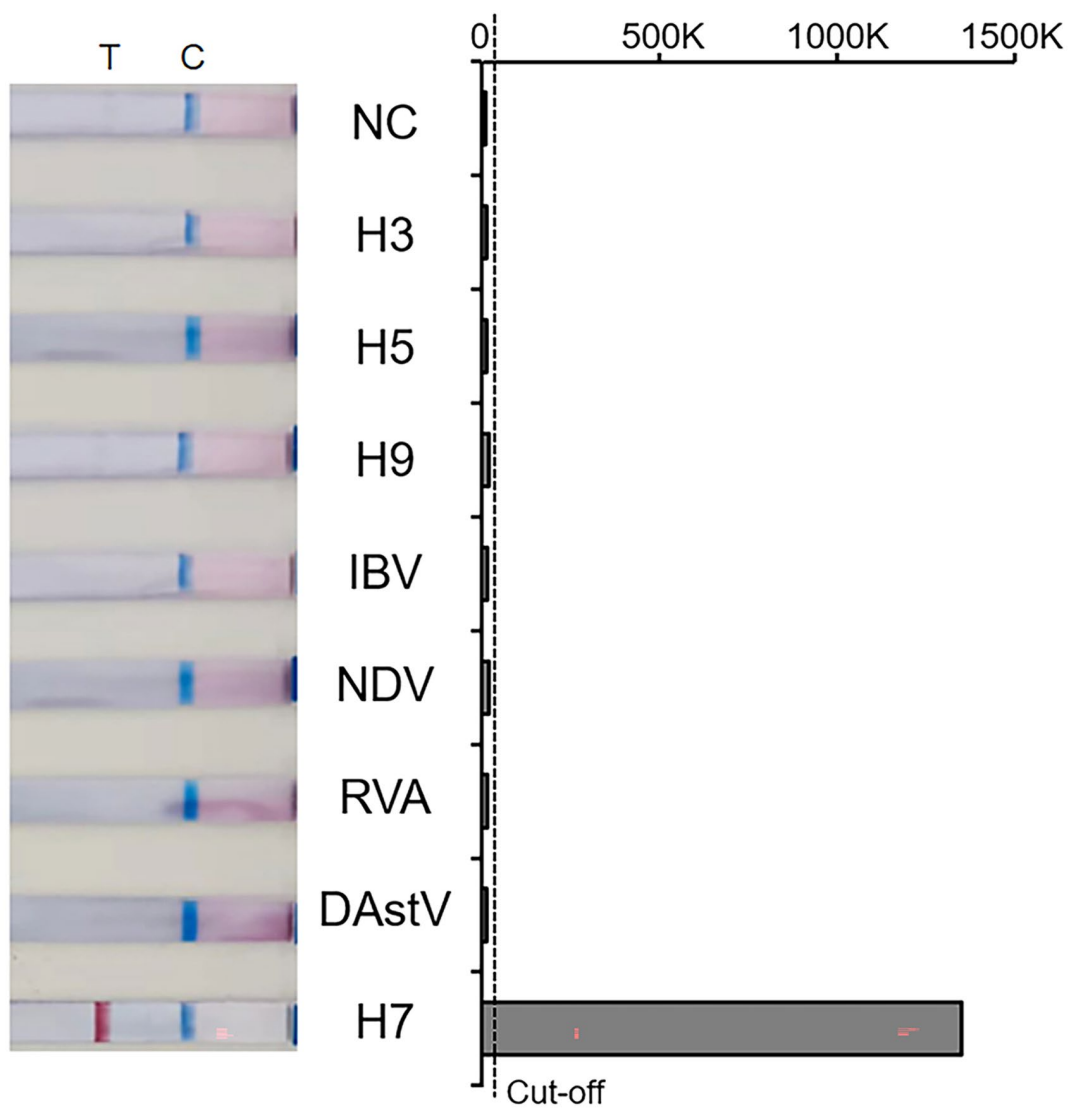
Amplification results of Specificity. T: Test line; C: Control line; NC: Negative control; 0–1,500 K: The optical density of the selected T and C lines; Cut-off: The critical value for determining the negativity and positivity of the result.

The specificity of the RT-RAA-LFD method was tests using nucleic acids of six kinds of viruses, including NDV and IBV. The result showed that all quality control lines were present, indicating validity of the results. And only the AIV-H7 strain showed a positive result at the detection line location, while the NTC and all other viruses tested produced no positive visual readout (Figure 3). These results above demonstrate that the RT-RAA-LFD method has excellent specificity for detecting AIV-H7.

### 3.4. The analytical sensitivity of the RT-RAA-LFD assay

**Figure 4:**
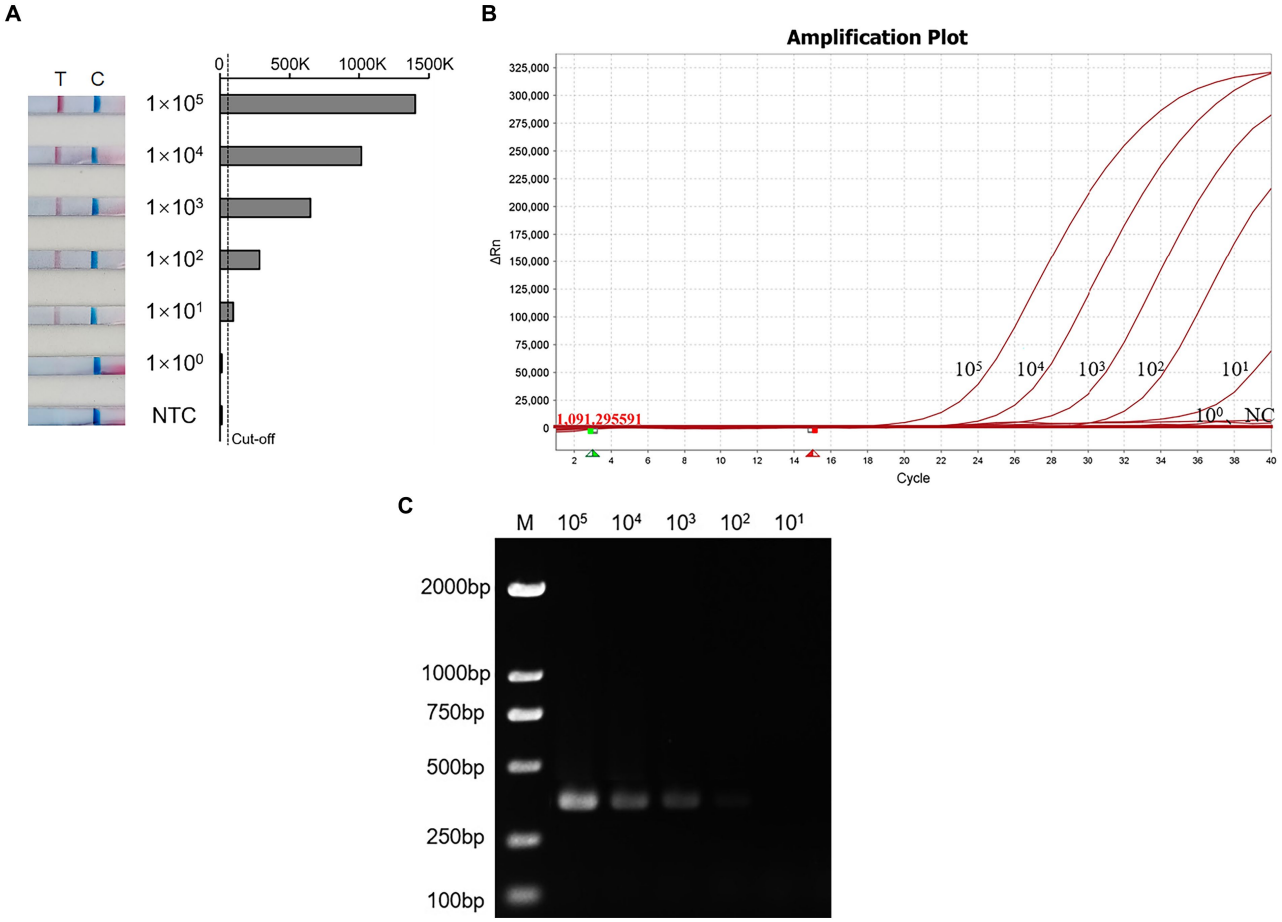
The sensitivity of the RT-RAA-LFD, RT-qPCR, and RT-PCR assay. **(A)** Sensitivity test of RT-RAA-LFD. T: Test line; C: Control line; NTC: Negative control; Cut-off: The critical value for determining the negativity and positivity of the result. **(B)** Sensitivity test of RT-qPCR. NC: Negative control; 10^5^–10^0^: Different dilution concentrations of standards. **(C)** Sensitivity test of RT-PCR. M: DL2000 Maker; 10^6^–10^0^: Different dilution concentrations of standards.

The sensitivity of the RT-PCR, RT-qPCR, and RT-RAA-LFD methods were evaluated using AIV-H7 standard diluted into different concentration gradients (ranging from 10^5^ copies/μL to 10^0^ copies/μL). As depicted in Figure 4A, strips appeared at all quality control line positions, and various shades of red lines corresponding to different concentrations of standards were observed at the detection line positions. The results of 8 repeated experiments demonstrated that 10^5^, 10^4^, and 10^3^ copies/μL were consistently positive in all 8 replicates, with 10^2^ copies/μL being positive in 5 out of 8 replicates. However, 10^1^ copies/μL only tested positive three times, while 10^0^ copies/μL were consistently negative. Therefore, the detection limit of the H7 RT-RAA-LFD method was 10^1^ copies/μL (Figure 4A and Table 3) the same as the RT-qPCR method (Figure 4B), whereas the detection limit of the RT-PCR method was 10 times lower (Figure 4C). The consistency results of the different kinds of methods makes our results more reliable.

**Table 3:** Minimum detection limit of this method.

Copies/μL	No. of positive samples/No. of samples tested by the RT-RAA-LFD assays for detection of AIV-H7
10^5^	8/8
10^4^	8/8
10^3^	8/8
10^2^	5/8
10^1^	3/8
10^0^	0/8

### 3.5. Detection of clinical samples

A total of 132 clinical samples collected from live poultry markets were tested for H7 using RT-RAA-LFD, RT-PCR, and RT-qPCR simultaneously. The results indicated that the positive rate of RT-RAA-LFD was 35.61% (47/132), while the positive rates for RT-PCR and RT-qPCR were 31.82% (42/132) and 35.61% (47/132), respectively. Compared to RT-PCR, the detection coincidence rate was 96.35, and 100% for both RT-RAA-LFD and RT-qPCR (Table 4). All 42 positive samples identified by RT-PCR were also positive by both RT-RAA-LFD and RT-qPCR methods. The kappa value of RT-RAA-LFD and RT-PCR was 0.9169 (*p <* 0.001) (Table 5). All of these results indicated that the RT-RAA-LFD assay developed in this study is suitable for the field rapid detection of AIV-H7 in clinical samples.

**Table 4:** Detection results of clinical samples using different detection methods (*n* = 132).

Methods	Positive cases	Negative cases	Positive rate (%)	Coincidence rate (%)
RT-RAA-LFD	47	85	35.61	–
RT-PCR	42	90	31.82	96.35
RT-qPCR	47	85	35.61	100

**Table 5:** The results of RT-RAA-LFD and RT-PCR assay by detecting clinical samples.

		RT-RAA-LFD		Total	Kappa (κ)	*p*-value of kappa
		Positive	Negative			
RT-PCR	Positive	42	0	42		
	Negative	5	90	95	0.9169	<0.001
Total		47	90	137		

## 4. Discussion

AIV is an important zoonotic pathogen that impacts wildlife, poultry, and human health (Webby and Webster, 2001). AIV can spread from wild birds to poultry, causing significant economic losses, and in some cases to humans, resulting in zoonotic infections (Hutchinson, 2018). The emergence of H7N9 caused five major waves of human infections in mainland China (Guinat et al., 2023), with the number of infections and deaths surpassing those caused globally by H5N1. Although H7 is comparatively less common in wild birds than H5, its broad avian host range may contribute to the AIV-H7 epidemic.

As a widely used method for the detection of variety pathogens, a number of methods have been proposed for AIV detection based on PCR. Jia developed a dual RT-PCR approach that can distinguish between highly pathogenic and low pathogenic H7 subtypes of AIV, with a minimum detection limit of 5.2 × 10^−3^ fg and 3.6 × 10^−1^ fg (Jia et al., 2017). RT-qPCR assays are a highly accurate and precise method for measuring transcript expression levels (Davis-Vogel, 2022). Cui developed a variety of fluorescent PCR assays for H3N2, H1N1, and H7N9 to detect and distinguish Flu A subtypes. Graaf established an RT-qPCR assays for differential diagnosis of HP and LP H7 subtypes (Graaf et al., 2017). However, these methods require professional instruments and long reaction times, which are difficult to meet the needs for rapid detection.

RT-LAMP, a commonly used constant temperature detection method, can amplify in a short time and produce color changes when coupled with a pH indicator. Multiplex LAMP methods (Zhang et al., 2013) have been developed, however, they are monitored either in real-time using endpoint analysis such as gel electrophoresis (Liang et al., 2010) or pyrosequencing (Liang et al., 2012), or by annealing curves (Mahony et al., 2013). These techniques necessitate specialized and complex instruments, limiting the scope and applicability of multiplex LAMP methods. Additionally, the LAMP method is susceptible to false positives due to insufficient closure of the reaction system. The RT-RAA-LFD method is a promising approach for rapid detection at a constant temperature, shortening the reaction time while ensuring specificity and sensitivity, and visualizing the results on the test strip. This method has a relatively low threshold for operation, is no longer limited to fixed instruments, and can be widely used in rapid detection in the early stage of virus infection. The assay established in this study can be used for the AIV-H7 detection of clinical samples with a sensitivity of 10^1^ copies/μL.

However, during the experiment, we found that different test strips have different absorption rates for liquids due to different batches, which can lead to different times required for color development. This variability should be taken into account when interpreting results. Additionally, since this method adds amplification products to the test strip for determination of results, contamination such as aerosols that may occur during the experiment, especially in the laboratory environment, needs to be considered to avoid false positives. Therefore, it is important to follow proper laboratory safety protocols to minimize the risk of contamination.

Of course, compared with other detection methods, the RT-RAA-LFD method has great advantages in terms of cost. This method does not require expensive temperature control or result analysis instruments. The reaction conditions can be met using metal baths or even water baths, and the results can be determined by visual observation. After some targeted optimization such as optimize the storage temperature of reagents and improve the stability of the test strip, this method may be more advantageous in on-site rapid detection.

In conclusion, the RT-RAA-LFD method has multiple advantages with respect to established methods and is a rapid and high-quality etiological detection. The development of this method can serve as a guideline for establishing various DNA and RNA detection methods for other pathogens.

## Data availability statement

The datasets presented in this study can be found in online repositories. The names of the repository/repositories and accession number(s) can be found in the article/Supplementary material.

## Ethics statement

The experimental protocol in this study followed the animal welfare regulations of the World Organization for Animal Health and the Animal Welfare Committee of the China Animal Health and Epidemiology Center (CAHEC). Permission for the collection of swab samples was granted by the China Ministry of Agriculture and Rural Affairs, CAHEC, a series of relevant veterinary sections of provincial and county governments, and relevant farm owners.

## Author contributions

FZ: Conceptualization, Data curation, Formal analysis, Investigation, Methodology, Resources, Software, Supervision, Visualization, Writing – original draft, Writing – review & editing. JS: Data curation, Formal analysis, Methodology, Software, Writing – review & editing. JLu: Data curation, Formal analysis, Methodology, Software, Writing – review & editing. XYi: Data curation, Formal analysis, Writing – review & editing. XYu: Data curation, Formal analysis, Writing – review & editing. WJ: Data curation, Formal analysis, Writing – review & editing. JLi: Resources, Writing – review & editing. LY: Funding acquisition, Resources, Writing – review & editing. GH: Resources, Writing – review & editing. HL: Funding acquisition, Project administration, Resources, Supervision, Writing – review & editing. YL: Funding acquisition, Project administration, Resources, Supervision, Writing – review & editing.
